# MicroRNA and mRNA Dysregulation in Astrocytes Infected with Zika Virus

**DOI:** 10.3390/v9100297

**Published:** 2017-10-14

**Authors:** Robert A. Kozak, Anna Majer, Mia J. Biondi, Sarah J. Medina, Lee W. Goneau, Babu V. Sajesh, Jessy A. Slota, Vanessa Zubach, Alberto Severini, David Safronetz, Shannon L. Hiebert, Daniel R. Beniac, Timothy F. Booth, Stephanie A. Booth, Gary P. Kobinger

**Affiliations:** 1 Department of Laboratory Medicine and Pathobiology, University of Toronto, Toronto, ON M5S 1A8, Canada; Rob.kozak@gmail.com; 2 Molecular Patho Biology, National Microbiology Laboratory, Public Health Agency of Canada, Winnipeg, MB R3E 3R2, Canada; anna.majer@canada.ca (A.M.); sarah.medina@canada.ca (S.J.M.); jessy.slota@canada.ca (J.A.S.); stephanie.booth@canada.ca (S.A.B.); 3 Viral Diseases Division, National Microbiology Laboratory, Public Health Agency of Canada, Winnipeg, MB R3E 3R2, Canada; shannon.hiebert@canada.ca (S.L.H.); daniel.beniac@canada.ca (D.R.B.); tim.booth@canada.ca (T.F.B.); 4 Special Pathogens Program, National Microbiology Laboratory, Public Health Agency of Canada; Winnipeg, MB R3E 3R2, Canada, mia.biondi@mail.mcgill.ca; 5 Medical Microbiology, Public Health Ontario Laboratory, Toronto, ON M5G 1M1, Canada; Lee.goneau@oahpp.ca; 6 Research Institute in Oncology and Hematology, Cancer Care Manitoba, Winnipeg, MB R3E 0V9, Canada; bsajesh@cancercare.mb.ca; 7 Viral Exanthemata and STD, National Microbiology Laboratory, Public Health Agency of Canada, Winnipeg, MB R3E 3R2, Canada; vanessa.zubach@canada.ca (V.Z.); alberto.severini@canada.ca (A.S.); 8 Viral Zoonoses, National Microbiology Laboratory, Public Health Agency of Canada, Winnipeg, MB R3E 3R2, Canada; david.safronetz@canada.ca; 9 Department of Pathology and Laboratory Medicine, University of Pennsylvania School of Medicine, Philadelphia, PA 19104, USA; 10 Infectious Diseases Research Centre, Université Laval, Quebec, QC G1V 4G2, Canada

**Keywords:** Zika virus, microRNA, human astrocytes, host response, pathogenesis, flavivirus

## Abstract

The Zika virus (ZIKV) epidemic is an ongoing public health concern. ZIKV is a flavivirus reported to be associated with microcephaly, and recent work in animal models demonstrates the ability of the virus to cross the placenta and affect fetal brain development. Recent findings suggest that the virus preferentially infects neural stem cells and thereby deregulates gene expression, cell cycle progression, and increases cell death. However, neuronal stem cells are not the only brain cells that are susceptible to ZIKV and infection of other brain cells may contribute to disease progression. Herein, we characterized ZIKV replication in astrocytes, and profiled temporal changes in host microRNAs (miRNAs) and transcriptomes during infection. We observed the deregulation of numerous processes known to be involved in flavivirus infection, including genes involved in the unfolded protein response pathway. Moreover, a number of miRNAs were upregulated, including miR-30e-3p, miR-30e-5p, and, miR-17-5p, which have been associated with other flavivirus infections. This study highlights potential miRNAs that may be of importance in ZIKV pathogenesis.

## 1. Introduction

Zika virus (ZIKV) is a mosquito-borne flavivirus that was initially discovered in Uganda in 1947, and subsequently spread throughout Africa and Asia [1,2]. Although historically understudied, due to its dramatic spread to Latin America, following outbreaks on Yap Island and in French Polynesia, it is increasingly recognized as a virus of public health concern with significant epidemic and pathogenic potential [3,4]. Recently, ZIKV has been linked with severe infectious complications, including congenital Zika virus syndrome (characterized by craniofacial disproportion, spasticity, seizures, brainstem dysfunction, and microcephaly), and Guillain–Barré syndrome (GBS) in Latin America and the Caribbean [5,6,7,8,9]. ZIKV is highly neurotropic, and has been demonstrated to impact the developing fetal brain in mouse infection models [10]. Although pathogenic mechanisms behind this are still being characterized, there seems to be a significant impact on cell cycle progression and differentiation of neurons.

It has been shown that ZIKV can infect numerous cell types, including dendritic cells, placental macrophages, and neural progenitor cells [11,12,13,14]. Additionally, ZIKV infection of organoid cultures has revealed the presence of the virus in glial cells [15]. Interestingly astrocytes are some of the first responders to viral infections, and have been shown to be susceptible to infection by both African and Asian strains of the virus [16]. ZIKV interaction with AXL receptor on human astrocytes and subsequent subversion of the host innate immune response has been suggested as an important contributing factor for pathogenesis [17]. Moreover, in vivo mouse studies indicate astrocytes are early targets of the virus, potentially due to their interaction with the vascular system, with ZIKV being detected in multiple brain regions as early as 4 days post-infection (DPI) [18]. Infection of these cells may play a role in the spread of the virus, as release of new progeny virus may promote subsequent neuronal infection [19]. Astrocytes are an important component of the central nervous system (CNS). Their functions are multifaceted, including mediation of blood–brain barrier integrity, repair of tissue damage, production of growth factors, support of neuronal function, interaction with microglia, and moderation of the innate and adaptive responses to pathogens [20]. Recent human cases of ZIKV infection have revealed the presence of diffuse astrogliosis, suggesting their involvement in the infectious process [6]. However, to date the effect of ZIKV infection on astrocyte microRNA (miRNA) expression has not been examined.

Flaviviruses moderate gene expression as a means of counteracting antiviral responses or maintaining a favorable environment for replication [21], a process that can involve altering the host miRNA profile. MiRNAs are a class of small non-coding RNAs that regulate gene expression at the post-transcriptional level [22,23]. They have been implicated in the regulation of numerous cellular pathways, including those associated with pathogenesis. Furthermore, deregulation of miRNA expression has been found in numerous vector-borne flaviviruses [24]. For example, miR-532-5p has been shown to act on host genes *SESTD1* and *TAB3* in HEK293T cells in order to promote an antiviral effect against the West Nile virus (WNV) [25]. Conversely, upregulation of both miR-30e and miR-146a during dengue virus (DENV) infection reportedly generates an inflammatory response that is favorable for the virus [26]. In vivo infection models have also suggested important roles of miRNA in regulating infection and brain development. The presence of Japanese encephalitis virus (JEV) increases the expression of miR-19b-3p in mouse microglial and human astrocyte cells, while inhibitors against this miRNA decrease cytokine production and increased survival in infected mice [27]. Additionally, borna disease virus infection modulated miRNA expression in neonatal rats, altering hippocampus development, cell differentiation, proliferation, and apoptosis [28]. However, no study to date has examined how ZIKV modulates cellular miRNA expression, despite the fact that miRNAs are involved in numerous viral infections including those caused by flaviviruses [23,29,30]. That being said, several groups have elucidated changes in gene expression profiles following ZIKV infection in human and mouse neuronal cells [11,12,15]. As such, understanding the interplay between miRNA expression and ZIKV infection is essential to further our understanding of the host pathways involved in pathogenesis. The present study demonstrates that a human astrocyte cell line, derived from human fetal glial cells, is susceptible to ZIKV infection, and that viral infection deregulates host transcriptomic profiles. Of particular interest is the identification of numerous miRNAs that had been associated previously with other flavivirus infections, including miR-30e-5p, miR-296-5p, and miR-431-5p [24]. Our study also shows that miR-17-5p is one of the most upregulated miRNAs, and it is a well-characterized miRNA in regulation of the unfolded protein response, a pathway important for flavivirus replication [31].

## 2. Methods

### 2.1. Virus and Cells

An SVG-derived cell line (SVG-A) was generously provided by Dr. Eugene O. Major and Dr. Michael W. Ferenczy at the Laboratory of Molecular Medicine and Neuroscience, National Institute of Neurological Disorders and Stroke, National Institutes of Health, Bethesda, Maryland. Cells were maintained in Dulbecco’s Modified Eagle Medium (Life Technologies, Burlington, ON, Canada), supplemented with 10% fetal bovine serum (Life Technologies), 2 mM l-glutamine, and 100 IU/mL penicillin/100 μg/mL streptomycin (Life Technologies). Vero cells were maintained in Dulbecco’s Modified Eagle Medium with 10% fetal bovine serum, 2 mM l-glutamine, and 100 IU/mL penicillin/100 μg/mL streptomycin. All infection studies used Zika virus (ZIKV) strain PRVABC59 (Genbank accession number KU501215.1) which was isolated from a patient in Puerto Rico in December 2015 [32]. To assess viral replication, cells were seeded into 6-well plates, and infected at a multiplicity of infection (MOI) of 1 for 1 h at 37 °C. Following this, the inoculum containing the virus was removed, cells were washed with phosphate-buffered saline (PBS), and fresh media was added. At predetermined time points the supernatant was removed and viral titer determined. The number of infectious viruses was determined by tissue culture infectious dose 50 (TCID_50_) according to established protocols [33]. Survival of cells after viral infection was determined by PrestoBlue^TM^ Cell Viability Reagent (Life Technologies), a resazurin dye-based metabolic assay. Cells were plated at concentrations of 1 × 10^4^ viable cells/well and allowed to adhere overnight. Cells were either uninfected or infected and at subsequent time points after viral infection PrestoBlue^TM^ Cell Viability Reagent was added according to the manufacturer’s protocol. Cell viability was determined by comparing fluorescence readings of infected cells to uninfected controls. All experiments were run in triplicate.

### 2.2. Immunofluorescence

Cells were infected with ZIKV and fixed with 4% paraformaldehyde (PFA)/sucrose for 10 min at room temperature at 48 and 82 h post-infection (hpi), and were processed as previously described [34]. Briefly, samples were permeabilized with 0.5% Triton X-100 (Sigma, Oakville, ON, Canada) in PBS for 10 min. Samples were then incubated with primary antibodies for 1 h at room temperature, washed once with 0.1% Triton X-100 and twice with PBS, and were then incubated with secondary antibodies for 1 h at room temperature. Samples were washed once with 0.1% Triton X-100 and twice with PBS, and mounted using ProLong Gold (Life Technologies). The following antibodies were used: anti-flavivirus group antigen antibody (mouse; 1:1000; gift from Rocky Mountain Laboratories), anti-glial fibrillary acidic protein (GFAP) antibody (rabbit; 1:500; Dako, Burlington, ON, Canada), anti-mouse secondary conjugated to Alexa-Fluor 647 (goat; 1:200; Life Technologies), and anti-rabbit secondary conjugated to Alexa-Fluor 488 (goat; 1:200; Abcam, Toronto, ON, Canada). Images were acquired using a Zeiss LSM 700 confocal microscope, visualized using Imaris (Bitplane, version 5, Concord, MA, USA) and panels were generated using Adobe Photoshop.

### 2.3. Electron Microscopy

Seventy-two hours post-infection, SVG cells were harvested in PBS and pelleted. Cell pellets were fixed with 2% paraformaldehyde/2.5% glutaraldehyde (Electron Microscopy Sciences, Hadfield, PA, USA) for 1 h at room temperature. Following fixation, excess fixative was removed, and pellets were mixed with liquid 3% Ultra-pure Low Melting Point Agarose (Life Technologies). Once solidified, cell/agarose pellets were cut into 1-mm pieces and added to tissue processing baskets. Cells were stained with 1% osmium tetraoxide, followed by a step-wise dehydration in acetone and then infiltration with Epon 812 resin (Electron Microscopy Sciences), completed in a Leica EM AMW automatic microwave tissue processor (Leica, Solms, Germany). Thin sectioning of cells was performed using the Leica Ultracut UCT microtome (Leica, Solms, Germany). Resin sections were then put onto 400 mesh copper grids and stained with 2% uranyl acetate, and 0.5% lead citrate. Sections were carbon-coated for stability in an Agar turbo carbon coater (Agar Scientific, Stansted, Essex, UK). Specimens were imaged in a FEI Tecnai F20 transmission electron microscope (TEM) operating at 200 kV, and at nominal instrument magnifications of 29,000× and 50,000×. Digital images of the specimens were acquired by an AMT Advantage XR 12 CCD camera (AMT, Danvers, MA, USA).

### 2.4. MicroRNA Next Generation Sequencing and Analysis

For miRNA and host transcriptome analysis, SVG-A cells were infected at an MOI = 0.1 plaque forming unit (PFU) and total RNA was harvested at various time points. Total RNA was isolated from cells using the Qiagen RNeasy kit according to manufacturer’s instructions (Qiagen, Toronto, ON, Canada). Next-generation sequencing (NGS) library preparation was done from 100–300 ng of total RNA using NEXTflex Small RNA-Seq kit v3 (BIOO Scientific, Austin, TX, USA) as per the manufacturer’s recommendations. Libraries were size selected via the Blue Pippin system (Sage Science, Beverly, MA, USA) by running the samples on a 3% agarose gel and isolating the 125–150 bp range followed by cleaning samples using PCRClean DX beads (Aline Biosciences, Woburn, MA, USA). Libraries were verified on a High Sensitivity DNA chip using the 2100 Bioanalyzer (Agilent, Santa Clara, California, USA). For analysis of miRNAs, libraries were size selected to enrich for constructs containing mature miRNAs. Sequencing was performed with the MiSeq v3 150 cycle kit (Illumina, San Diego, CA, USA) and 65 single-read cycles were used. Raw sequences were analyzed using the exceRpt small RNA-seq Pipeline v.4.3.3 by Genboree [35,36], using default settings (allowing for 1 mismatch) and removing the four random bases from both the -5p and -3p ends. All miRNAs containing less than 20 reads per million (RPM) in all control and at 24 and 48 hpi, were removed from subsequent analyses.

Bioinformatic prediction of possible biological processes regulated by miRNAs that were induced during ZIKV infection was performed using DIANA-miRPath v3.0 [37]. MiRNA gene targets previously experimentally validated were identified from the DIANA-TarBase v7.0 (*p*-value threshold of 0.05) database for each upregulated miRNA [38]. These targets were then filtered using the gene expression data identified for that time point and only genes that were downregulated by more than or equal to 2-fold were included in the final analysis. The final list of downregulated miRNA targets was then merged using the gene union analysis option with a false discovery rate correction (Benjamini and Hochberg) and a *p*-value threshold of 0.05. The Gene Ontology analysis was performed to identify biological processes that were potentially regulated by these miRNAs. Complete data sets are available through NCBI (reference number GSE89915).

### 2.5. Microarray Gene Expression Profiling and Analysis

Global gene expression profiling was determined as previously described [39] using whole human genome 4 × 44 k arrays (Agilent Technologies). Between 30 and 100 ng of pooled total RNA was used for two rounds of amplification. Feature Extraction version 11.5.1.1 was used to process the data files. Gene Ontology analyses were performed using QIAGEN’s Ingenuity Pathway Analysis (IPA, QIAGEN Redwood City, www.qiagen.com/ingenuity) where the log ratio cutoff was set to 0.3 and *p*-value was set to 1 × 10^6^. Raw data was deposited in Gene Expression Omnibus (GSE89915). We used DIANA-miRPath v3.0 to perform this analysis and identify the gene ontology biological processes that are enriched by the target genes at each time point post-infection. Gene targets for each upregulated miRNA (>2-fold) were identified using TarBase v7.0, a database that contains experimentally tested miRNA targets. We further filtered this list of genes with genes we identified to be downregulated by more than 2-fold in ZIKV infected astrocytes. This allowed us to obtain a more specific list of targets that were potentially regulated by miRNAs during ZIKV infection. We combined targets for each miRNA that were upregulated at each time post-infection and DIANA-miRPath v3.0 was used to identify the Gene Ontology networks that were significantly enriched by those genes.

### 2.6. Quantitative RT-PCR

Select miRNAs were further validated using individual miRCURY LNA Universal RT microRNA PCR (Exiqon, Vedbaek, Denmark). Briefly, cDNA was generated following manufacturer’s recommendations with 200 ng total RNA as input. Real-time PCR was performed as recommended by the manufacturer using the ExiLENT SYBR Green master mix (Exiqon). Data was normalized to the C_T_ values of U6 and analyzed as described below.

Select mRNAs were further validated using individual TaqMan Gene Expression Assays (ThermoFisher Scientific, Burlington, ON, Canada). Briefly, total RNA was reverse transcribed using the High Capacity cDNA Reverse Transcription kit (ThermoFisher Scientific), purified via the ChargeSwitch PCR Clean-Up Kit (ThermoFisher Scientific) and 50 ng of cDNA was used for each individual real-time PCR reaction as per the manufacturer’s recommendation. We used TaqMan Fast Universal PCR Master Mix (2×), No AmpErase UNG (ThermoFisher Scientific). Data was normalized to the C_T_ values of GAPDH and analyzed using the 2-ΔΔC_T_ method. Data is represented as mean ± standard deviation calculated from three biological replicates.

### 2.7. Statistical Analysis

Figures were generated using GraphPad Prism 6.0 software (La Jolla, CA, USA). Statistical analyses for qRT-PCR were also performed using this software where a 2-tailed, paired Student’s *t*-test was used and a *p*-value of <0.05 defined significance.

## 3. Results

### 3.1. Astrocytes Support ZIKV Replication

**Figure 1:**
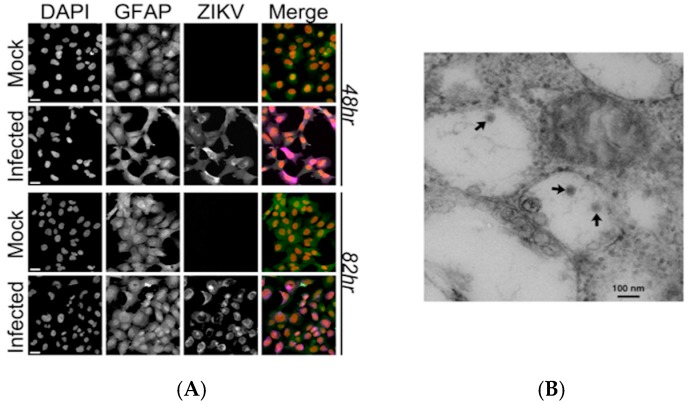
Zika virus (ZIKV) detected in a human astrocyte cell line. (**A**) Confocal images of SVG-A cells infected with ZIKV after 48 and 82 h post-infection (hpi) for the ZIKV envelope protein (ZIKV: purple), 4’, 6-Diamidine-2’-phenylindole dihydrochloride (DAPI: red) and glial fibrillary acidic protein (GFAP: green). Infection was performed in triplicate and one representative image is shown. Scale bar = 20 µm; (**B**) A representative electron micrograph of the negative stained viral particles within intracellular vesicles at 48 hpi (multiplicity of infection, MOI = 1 PFU). Scale bar = 100 nm.

Astrocytes are an early target of ZIKV infection, and astrogliosis has been reported in human cases of microcephaly following ZIKV infection [6]; this suggests that astrocytes might play a role in ZIKV pathogenesis. To evaluate this, we examined viral replication in the human fetal astrocyte cell line, SVG-A [40]. Cells were infected with a contemporary ZIKV strain, isolated from a clinical case in Puerto Rico at a MOI of 1 PFU. Immunofluorescent staining of these cells with an antibody against flaviviruses indicated the presence of ZIKV within the cells, confirming active infection, and viral proteins were detected in both the cytoplasm and in the perinuclear space. Subsequent analysis by electron microscopy identified intracellular viral particles with the morphological characteristics of flaviviruses (Figure 1).

**Figure 2:**
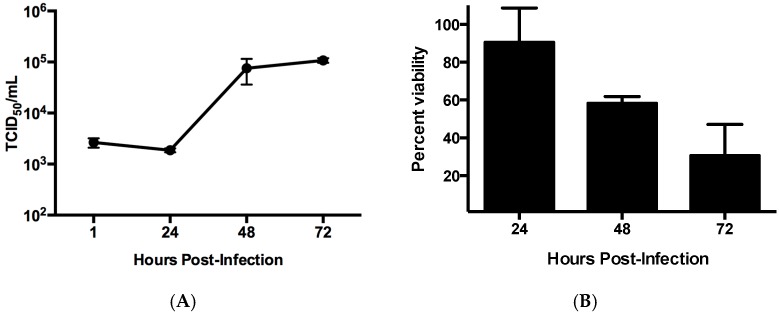
Viral replication in SVG-A cells. (**A**) Cells were infected at a multiplicity of infection (MOI) of 1 PFU and at various time points the titers were determined by TCID_50_ in order to establish a one-step growth curve; (**B**) Cell viability was determined using a resazurin-based assay in cells infected at an MOI = 1 PFU. All experiments were performed in triplicate. Error bars represent standard deviation.

Replication of ZIKV in SVG-A cells was evaluated through a single step growth curve (Figure 2A). Productive infection in these cells yielded titers of 10^5^ TCID_50_/mL by 72 hpi. Analysis of cell viability was performed using a resazurin-based dye that demonstrated an increase in cell death as the infection progressed (Figure 2B). Collectively, this data confirmed that SVG-A cells are susceptible to ZIKV infection and can support viral propagation.

### 3.2. ZIKV Infection Alters Cellular miRNA Expression

**Figure 3:**
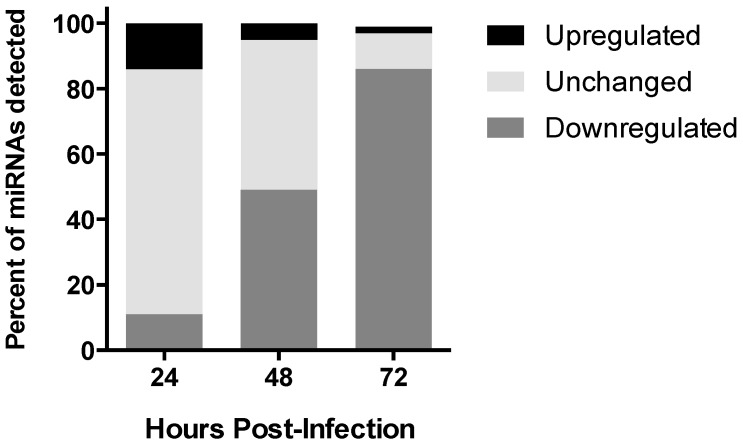
Deregulation of miRNA expression during ZIKV infection of astrocytes. Global deregulation of miRNA expression post-infection over time.

MicroRNAs are key regulators in cellular processes, and are disrupted following flavivirus infection [41]. In order to investigate the effect of ZIKV on miRNA expression we performed deep sequencing of small RNAs isolated from infected SVG-A cells at several time points following infection, and compared this to uninfected cells. Both infected and uninfected cell libraries yielded in excess of 2.2 million sequence reads. These sequence reads were mapped to the human genome, resulting in 1.3–2.2 million unique mappable sequences per condition. At 24 hpi, 11 miRNAs were upregulated by more than 2-fold. At 48 hpi, only miR-411-3p, miR-323a-5p, and miR-194-5p were upregulated by more than 2-fold and at 72 hpi, miR-9-5p was induced over 7-fold. The miRNAs with the highest induction during the course of infection were miRNA-9-5p (7.2-fold), miR-17-5p (6-fold), miR-146b-5p (3.8-fold), and miR-30e-3p (3.3-fold) (Table 1 and Table S1). In order to validate the regulated miRNAs, a subset was selected, and the changes in expression level for miR-17-5p, miR-30e-3p, miR-30e-5p, and miR-744 were validated using qRT-PCR (Figure S1). Together, this data indicates that ZIKV changes the abundance of specific miRNAs during the infection (Table 1). Overall, our findings suggest that there was a global trend towards a decrease in miRNA expression in ZIKA-infected cells over the course of the infection compared to uninfected controls (Figure 3). However, the subset of miRNAs increased in abundance by ≥2.0-fold coincident with infection, and was the focus of further analysis.

### 3.3. Cellular Gene Expression Is Altered by ZIKV Infection

**Figure 4:**
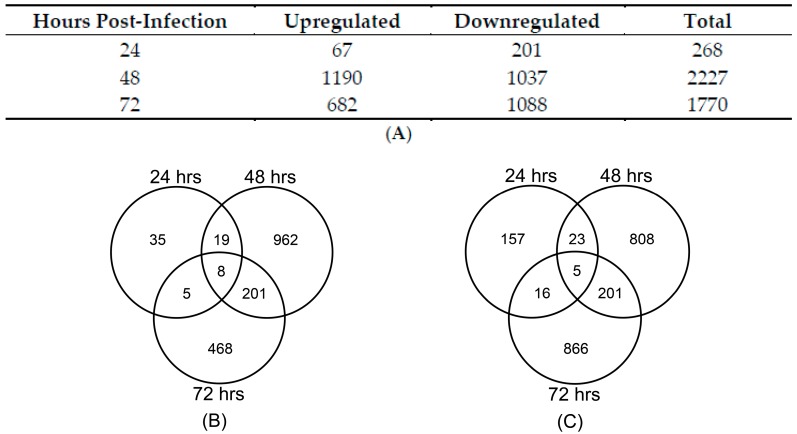
Changes in cellular gene expression during ZIKV infection. (**A**) List of deregulated genes identified at each time point in astrocytes infected with ZIKV at an MOI = 0.1 PFU. Genes deregulated by more than 2-fold (*p*-value < 0.001) are listed. Number of genes found to be commonly deregulated between the various time points when only (**B**) upregulated or (**C**) downregulated genes were compared.

**Figure 5:**
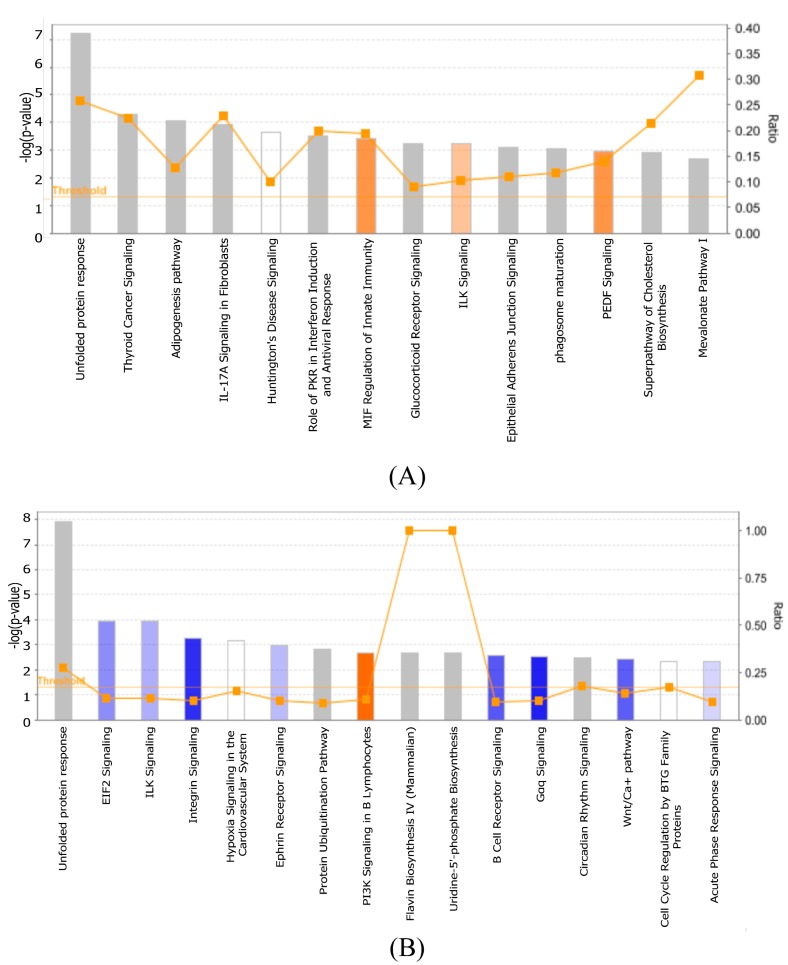
Deregulation of biological process during ZIka virus (ZIKV) infection. (**A**) 48 hpi and (**B**) 72 hpi of the top canonical pathways that were deregulated during ZIKV infection of SVG cells. The color of the bars indicates predicted pathway activation based on z-score (orange = activation; blue = inhibition; gray = no prediction can be made; white = z-score close to 0). Orange line represents the ratio = # genes in dataset/total # of genes that compose that pathway. The horizontal yellow line indicates the p-value threshold. Fisher’s exact test, right-tailed, was used to calculate negative log of *p*-value.

Next we investigated the transcriptional response to ZIKV with the aim of identifying potential miRNA regulated gene expression networks at 24, 48, and 72 hpi via whole genome microarrays. Gene expression changes were identified using the criteria of greater than 2-fold and a *p*-value of <0.001 (Figure 4A). Validation of select gene expression was also performed using qPCR (Figure S2). *In silico* analysis, using Ingenuity pathway analysis (IPA) identified differentially expressed genes that are enriched in particular cellular pathways following ZIKV infection. The pathways that were identified at 24, 48, and 72 hpi are shown in Table 2. Interestingly, while the gene expression profiles throughout the time course of infection differed between time points, genes within the unfolded protein response pathway showed upregulation at both 48 and 72 hpi (Figure 5).

### 3.4. ZIKV Induces Modest Antiviral Response in Astrocytes

Previous groups have shown that ZIKV infection of human neuroprogenitor cells (hNPs) causes modest immune response [42]. Our data demonstrated similar findings whereby the increased expression of genes involved in the host protective response to viral infection was transient. Although there was activation of the genes encoding IFIT2 and OASL, both of which have been shown to play a role in restricting replication of flaviviruses [43,44], as well as the pattern recognition receptor gene *DHX58*, there were also many antiviral genes that were downregulated. Notably, similar to what has been reported in ZIKV infection of microglial cells, *IL1B* was downregulated at 48 hpi [45]. Additionally, there was no change in expression of other RIG-I like receptors, such as *DDX58 (RIG-I)* or *IFIH1*. Interestingly, *TLR3*, was downregulated at 48 hpi, which is in contrast to the findings of Dang and colleagues, who noted increased expression levels in cerebral organoids [46]. Biological processes that are perturbed following infection are illustrated in Figure 5, which shows the deregulation of genes involved in the activation of the interferon regulatory factors (IRFs) by cytosolic pattern receptors at 48 and 72 hpi. Overall, the expression profiles of these cells during ZIKV infection suggest a modest and transient antiviral response in astrocytes.

### 3.5. Predicted Genes Involved in ZIKV Cell Metabolism

**Figure 6:**
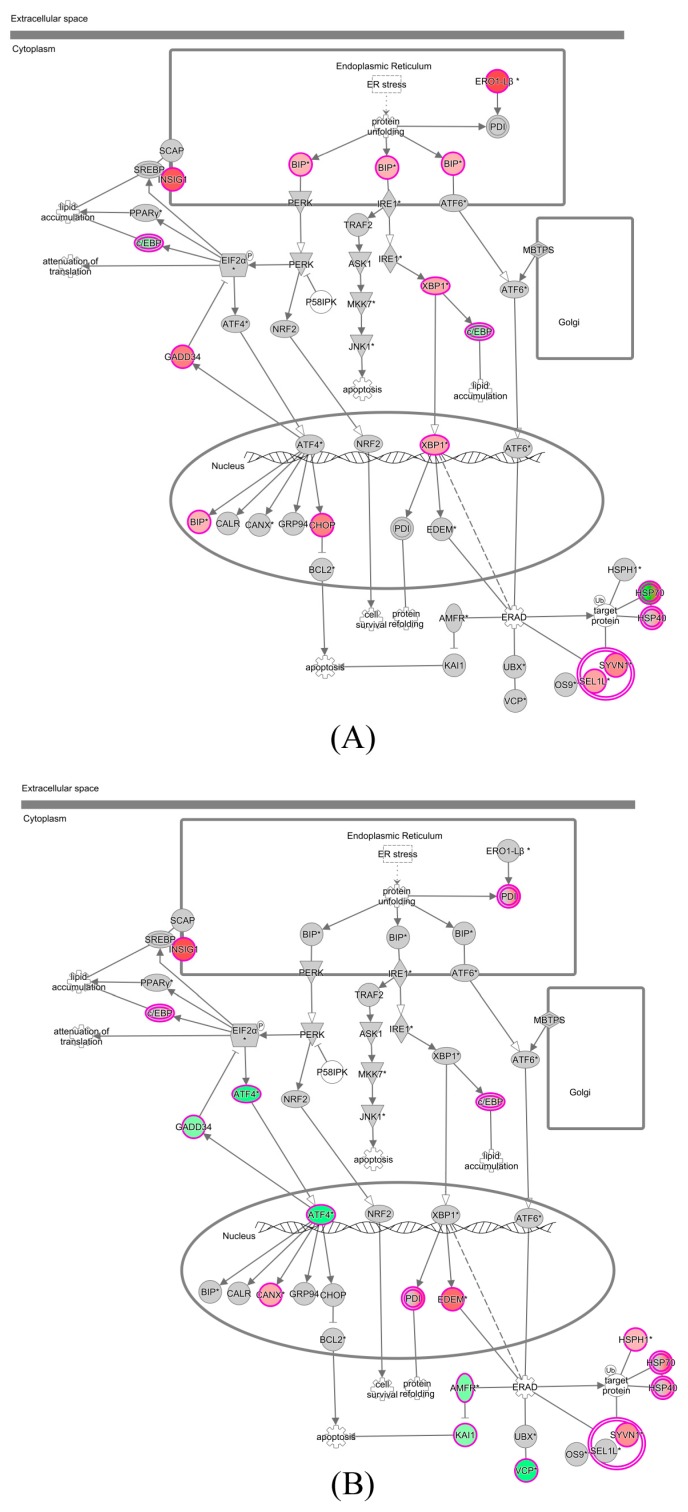
Predicted pathways deregulated during ZIKV infection. The unfolded protein response pathway is shown at (**A**) 48 hpi and at (**B**) 72 hpi. The pathway generated using the Ingenuity software package. The color indicates deregulation of that gene in infected as compared to control samples (red = upregulated; green = downregulated; grey = unchanged). White arrows = translocation; black arrows = direct interaction resulting in activation/expression/modification/transcription; dotted lines = indirect interaction.

IPA identified the unfolded protein response (UPR) pathway as being the most consistently altered over the time course of infection. In SVG-A cells, ZIKV infection stimulates the expression of *BiP* and *XBP1* that activate major arms of the UPR (Figure 6).

In addition, significant induction of expression of the genes coding for apoptosis effectors CHOP and GADD34, whose association with the phosphatase PP1 leads to the dephosphorylation of eIF2α, was apparent at 48 hpi. This corresponded with the decrease in cell viability in ZIKV-infected cells noted earlier (Figure 2). Numerous genes involved in RNA metabolism were also deregulated at 48 and/or 72 hpi. These included *DICER1*, the gene that codes a ribonuclease essential for the RNA interference pathway to produce the active small RNA component that represses gene expression, which was downregulated at 72 hpi, corresponding to the overall downregulation of miRNA expression. This a mechanism which has recently been described during DENV infection of A549 cells [47]. Of the genes that were highly induced, we observed an increase in expression of the glial cell line-derived neurotrophic factor (GDNF), which promotes the growth of neurons and neurites. Expression of neuronal growth factor (*NGF*) is also increased after 48 hpi. In addition to supporting neuronal growth, *NGF* has also been linked to the growth and differentiation of human B-lymphocytes and the differentiation of CD34+ hematopoietic progenitor cells, potentially serving as a link between the nervous and immune systems [48]. This suggests that genes involved in the cell cycle and innate immune response may be regulated by cellular miRNAs.

### 3.6. miRNA Deregulation Potentially Affects Cellular Gene Expression

**Figure 7:**
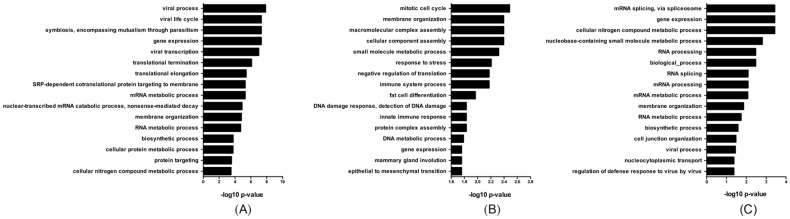
MiRNAs may regulate host genes involved in ZIKV infection. Biological gene ontology processes potentially regulated by at least one upregulated miRNA at (**A**) 24, (**B**) 48, and (**C**) 72 hpi.

We identified numerous targets for the upregulated miRNAs noted previously (Table 1), and performed a bioinformatic analysis to correlate biological processes that are potentially regulated by miRNAs during ZIKV infection of astrocytes (Table S2). Our analysis was limited to experimentally validated gene targets in order to maximize detection of biologically relevant functions. From this, genes involved in viral process, viral life cycle, and viral transcription were in the top processes identified at 24 hpi (Figure 7A), and nine miRNAs that were upregulated at this time point, including miR-17-5p, targeted 14 genes within these processes. Genes within these pathways were downregulated at that time point in infection (Table 3 and Table S2). At 48 hpi, we found that the putative targets of these miRNA are predicted to be involved in the cell cycle, innate immune response, and DNA damage and response to stress (Figure 7B). Only miR-9-5p was upregulated at 72 hpi, and we found that this miRNA could potentially downregulate genes involved in mRNA processing, gene expression, and viral processes (Figure 7C and Table 3). Collectively, this data could provide evidence that specific miRNAs may regulate genes involved in several biological processes throughout infection including viral production, innate immune response and cell cycle, all of which are likely involved in ZIKV pathogenesis.

## 4. Discussion

In this study we used next-generation sequencing to identify miRNAs that are dysregulated in astrocytes during the course of ZIKV infection. Our findings suggest that ZIKV induces global downregulation of miRNAs with only a small subset being upregulated. Using bioinformatic analysis coupled with gene expression profiling, we correlated the highly expressed miRNAs with various host pathways involved in ZIKV infection and suggest these miRNAs may be playing a role in their regulation. Several groups have elucidated the gene expression profiles following ZIKV infection in human and mouse neuronal cells, however, changes in miRNA expression in cells of the astrocyte lineage have not been described [11,12,15]. Our findings suggest that the unfolded protein response pathway is a major target for modification by the virus. We also noted a decrease in cell viability in our resazurin-dye assay, further supporting this hypothesis. In addition, significant induction of expression of the genes coding for the autophagy associated factors CHOP and GADD34 was apparent at 48 hpi.

The unfolded protein response pathway is a cellular quality control mechanism that ensures that only properly folded proteins exit from the endoplasmic reticulum (ER) and accumulation of misfolded proteins trigger ER stress and autophagy, a target of many flaviviruses to facilitate viral replication and persistence. An example of this is seen with JEV, where upregulation of this pathway has been shown to benefit viral replication by degrading host RNA transcripts without affecting viral RNA [49]. Additionally, a recent report by Liang and colleagues demonstrates that ZIKV usurps components of the unfolded protein response and autophagy pathways through the NS4A-NS4B protein to potentially create membrane structures that serve as a replication site [50]. Furthermore, a screen of FDA approved compounds identified sorafenib, which targets the ER, as having anti-ZIKV activity [51]. Interestingly, one miRNA with a high level of expression was miR-17-5p, which has been linked to regulation of the unfolded protein response [52].

Many of the miRNAs that were upregulated during ZIKV infection of astrocytes were also previously identified to play a role in the pathogenesis of other flaviviruses. MiR-431-5p has been suggested to play a role in restricting HCV replication [21]. The miRNA species miR-30e-5p is upregulated by infection with influenza, DENV, and in some cases chronic HCV [26,53,54]. In the case of DENV, over-expression of both miR-431-5p and miR-30e-3p results in interferon (IFN)-β upregulation and activation of interferon-stimulated genes [26]. This suggests that both miR-431-5p and miR-30e-3p are possibly important for both neurological development and innate immunity. Using *in silico* methods, we predict that at 48 hpi, induced miRNAs may modulate genes involved in the cell cycle and innate immune response. Previous studies have found that ZIKV infection results in downregulation of genes involved in the cell cycle [12], a process that may be regulated by miRNAs such as miR-411-3p, miR-194-5p, and miR-323a-5p. One of these cell cycle-related genes, *CENPF*, is a kinetochore protein that is important for proper alignment of chromosomes during mitosis, and recruits additional genes that together are important for spindle-pore formation. Genes involved in centrosome maturation and spindle-pore formation have been implicated in microcephaly phenotypes [55]. In fact, previous studies identified that depletion of CENPF resulted in mitotic delay and mutations within the gene have been linked to microcephaly [56,57]. Perhaps, miR-194-5p could contribute to mitotic delay during ZIKV infection by inhibiting the expression of *CENPF*. In addition, innate immune response genes *CD59* and *PTX3* were both downregulated and potentially inhibited by miRNAs miR-194-5p and miR-411-3p. Both genes are involved in complement activation and function and may result in decreasing immune activation during infection. Furthermore, we found that *DICER1* transcripts were downregulated at 72 hpi, which could have contributed to the overall global downregulation of miRNAs, although further experiments would be required to demonstrate this. A decrease in expression of *DICER* has also been observed after infection of cells with DENV [47] and resulted in enhanced viral replication. It is possible Zika virus infection of SVG cells also uses a similar mechanism to downregulate *DICER*, which in turn globally shuts down miRNA expression and aids viral replication. Collectively, this data could suggest that the miRNA profile modulated by ZIKV involves both miRNA species related to neurological development and the innate immune response to flaviviruses.

The induction of expression of relatively few pro-inflammatory genes may reflect a requirement for the activation of astrocytes by cytokines produced from other immune cells during infection, possibly brain microglia. A recent reported case of microcephaly described only mild focal inflammation in the brain, suggesting ZIKV does not necessarily induce a robust pro-inflammatory response [6]. In our study, *DHX58*, which encodes LGP2, was consistently upregulated in response to ZIKV and overexpression of LGP2 has also been linked to inhibition of interferon induction [58]. The production of IFN within astrocytes was postulated to be due to the varied stimuli that differ between viruses. These include cytoplasmic detection of viral RNA via RIG-I like receptors, TLR signaling triggered by viral proteins, or by cell damage associated nucleic acids that are released by cells damaged during infection [59,60,61,62]. In the case of ZIKV infection, any or all of these scenarios are possible avenues for induction. Another gene linked to flavivirus infection that has a role in inflammation and pathological processes is the enzyme prostaglandin-endoperoxide synthase 2 (*PTGS2*), otherwise known as *COX-2*, which was significantly induced throughout the time-course of infection. PTGS2 is able to induce chemotactic cytokines, mediators of blood–brain barrier disruption such as plasminogen activator proteins, apoptotic death, and activation of microglia [63,64].

At present, 70% of currently identified miRNAs are expressed in the brain [65] Therefore, the exploitation of these by a neurotropic virus, such as ZIKV, to facilitate replication and pathogenesis is not surprising. That being said, we acknowledge that one major limitation of our study is the further need for a comprehensive understanding of the interplay between miRNA, host gene expression, and viral pathogenesis. Therefore, additional studies, including those comparing the response in other neuronal cells such as microglia, are required to determine what role they may play and how they influence phenotypic changes in ZIKV-infected individuals.

## Figures and Tables

**Table 1:** List of upregulated microRNAs (miRNAs).

Hours Post-Infection	miRNA ID	Fold Change
24	hsa-miR-17-5p	6.0
	hsa-miR-146b-5p	3.8
	hsa-miR-30e-3p	3.3
	hsa-miR-296-5p	2.6
	hsa-miR-1303	2.6
	hsa-miR-4521	2.5
	hsa-miR-30e-5p	2.4
	hsa-miR-107	2.2
	hsa-miR-431-5p	2.1
	hsa-miR-7-5p	2.1
	hsa-miR-361-3p	2.0
48	hsa-miR-411-3p	2.7
	hsa-miR-323a-5p	2.6
	hsa-miR-194-5p	2.0
72	hsa-miR-9-5p	7.2

**Table 2:** Top pathways enriched in upregulated genes identified at 24, 48, and 72 hpi.

Hours Post-Infection	Biological Process/Pathway	*p*-Value	Genes
24	p53 signaling	5.31 × 10^−3^	3
	Ephrin B signaling	6.34 × 10^−3^	3
48	Unfolded protein response	3.08 × 10^−10^	18
	Hypoxia signaling in the cardiovascular system	5.81 × 10^−8^	17
	Huntington’s disease signaling	1.73 × 10^−7^	35
	ILK signaling	4.13 × 10^−7^	30
	Role of IL-17A in arthritis	5.5 × 10^−7^	11
	NRF2-mediated oxidative stress response	4.26 × 10^−6^	27
	Neurotrophin/TRK signaling	4.45 × 10^−5^	15
	Glucocorticoid receptor signaling	5.63 × 10^−5^	34
	Activation of IRF by cytosolic pattern recognition receptors	5.72 × 10^−5^	13
	Dendritic cell maturation	7.78 × 10^−5^	25
72	Unfolded protein response	8.7 × 10^−9^	13
	ILK signaling	1.43 × 10^−5^	19
	Ephrin receptor signaling	4.05 × 10^−4^	15
	Aldosterone signaling in epithelial cells	7.75 × 10^−4^	14
	ERK/MAPK signaling	1.61 × 10^−3^	15
	Huntington’s disease signaling	1.7 × 10^−3^	17
	eNOS signaling	1.84 × 10^−3^	12
	Hypoxia signaling in the cardiovascular system	1.87 × 10^−3^	18
	Glucocorticoid receptor signaling	1.89 ×10^−3^	8
	JAK/Stat signaling	1.89 × 10^−3^	8

**Table 3:** MicroRNA targets identified for each gene ontology annotation.

Hours Post-Infection	Gene Ontology	miRNA ID	# Gene Targets	Gene ID
24	Viral Process	hsa-miR-17-5p	8	*B2M, RPSA, RPL21, RPS11, SUPT16H, RPL19, CHMP3, CBX5*
		hsa-miR-30e-3p	5	*B2M, YWHAE, RPL23, SUPT16H, CBX5*
		hsa-miR-107	6	*B2M, RPSA, RPS25, CREBBP, CHMP3, CBX5*
		hsa-miR-7-5p	5	*B2M, YWHAE, RPL23, PSMB7, CBX5*
		hsa-miR-361-3p	1	*B2M*
		hsa-miR-30e-5p	3	*RPL38, CHMP3, CBX5*
		hsa-miR-4521	1	*RPL23*
		hsa-miR-146b-5p	1	*SUPT16H*
		hsa-miR-1303	2	*SUPT16H, CBX5*
	Viral Life Cycle	hsa-miR-107	3	*RPS25, RPSA, CHMP3*
		hsa-miR-17-5p	5	*RPSA, RPL21, RPS11, RPL19, CHMP3*
		hsa-miR-30e-5p	2	*RPL38, CHMP3*
		hsa-miR-30e-3p	1	*RPL23*
		hsa-miR-4521	1	*RPL23*
		hsa-miR-7-5p	1	*RPL23*
	Viral Transcription	hsa-miR-107	2	*RPS25, RPSA*
		hsa-miR-17-5p	4	*RPSA, RPL21, RPS11, RPL19*
		hsa-miR-30e-5p	1	*RPL38*
		hsa-miR-30e-3p	1	*RPL23*
		hsa-miR-4521	1	*RPL23*
		hsa-miR-7-5p	1	*RPL23*
48	Cell Cycle	hsa-miR-411-3p	1	*PPP1CC*
		hsa-miR-194-5p	6	*NUP107, PSME3, RRM2, ARPP19, CENPF, DYNC1H1*
		hsa-miR-323a-5p	1	*DYNC1H1*
	Immune System Response	hsa-miR-411-3p	5	*NCKAP1, PRKAR1A, TAB3, CD59, GSK3B*
		hsa-miR-194-5p	7	*PSME3, PTX3, RAB35, TNRC6A, ACTG1, CAV1, DYNC1H1*
		hsa-miR-323a-5p	2	*DYNC1H1, CD44*
	Innate Immune Response	hsa-miR-411-3p	5	*NCKAP1, PRKAR1A, TAB3, CD59, GSK3B*
		hsa-miR-194-5p	3	*ACTG1, TNRC6A, PTX3*
72	Viral Process	hsa-miR-9-5p	6	*NUP214, RPL6, AP1G1, AP2M1, KLC1, KPNB1*

## Supplementary Materials

Supplementary materials can be found at www.mdpi.com/1999-4915/9/10/297/s1.

## References

[1] Dick G.W., Kitchen S.F., Haddow A.J. (1952). Zika virus. I. Isolations and serological specificity. Trans. R. Soc. Trop. Med. Hyg..

[2] Faye O., Freire C.C., Iamarino A., Faye O., de Oliveira J.V., Diallo M., Zanotto P.M., Sall A.A. (2014). Molecular evolution of Zika virus during its emergence in the 20(th) century. PLoS Negl. Trop. Dis..

[3] Duffy M.R., Chen T.H., Hancock W.T., Powers A.M., Kool J.L., Lanciotti R.S., Pretrick M., Marfel M., Holzbauer S., Dubray C. (2009). Zika virus outbreak on Yap Island, Federated States of Micronesia. N. Engl. J. Med..

[4] Besnard M., Eyrolle-Guignot D., Guillemette-Artur P., Lastere S., Bost-Bezeaud F., Marcelis L., Abadie V., Garel C., Moutard M.L., Jouannic J.M. (2016). Congenital cerebral malformations and dysfunction in fetuses and newborns following the 2013 to 2014 Zika virus epidemic in French Polynesia. Euro. Surveill..

[5] Heymann D.L., Hodgson A., Sall A.A., Freedman D.O., Staples J.E., Althabe F., Baruah K., Mahmud G., Kandun N., Vasconcelos P.F. (2016). Zika virus and microcephaly: Why is this situation a PHEIC?. Lancet.

[6] Mlakar J., Korva M., Tul N., Popovic M., Poljsak-Prijatelj M., Mraz J., Kolenc M., Resman Rus K., Vesnaver Vipotnik T., Fabjan Vodusek V. (2016). Zika virus associated with microcephaly. N. Engl. J. Med..

[7] Cauchemez S., Besnard M., Bompard P., Dub T., Guillemette-Artur P., Eyrolle-Guignot D., Salje H., van Kerkhove M.D., Abadie V., Garel C. (2016). Association between Zika virus and microcephaly in French Polynesia, 2013–2015: A retrospective study. Lancet.

[8] Oliveira D.B., Almeida F.J., Durigon E.L., Mendes E.A., Braconi C.T., Marchetti I., Andreata-Santos R., Cunha M.P., Alves R.P., Pereira L.R. (2016). Prolonged shedding of Zika virus associated with congenital infection. N. Engl. J. Med..

[9] Parra B., Lizarazo J., Jimenez-Arango J.A., Zea-Vera A.F., Gonzalez-Manrique G., Vargas J., Angarita J.A., Zuniga G., Lopez-Gonzalez R., Beltran C.L. (2016). Guillain-barre syndrome associated with Zika virus infection in Colombia. N. Engl. J. Med..

[10] Miner J.J., Cao B., Govero J., Smith A.M., Fernandez E., Cabrera O.H., Garber C., Noll M., Klein R.S., Noguchi K.K. (2016). Zika virus infection during pregnancy in mice causes placental damage and fetal demise. Cell.

[11] Nowakowski T.J., Pollen A.A., Di Lullo E., Sandoval-Espinosa C., Bershteyn M., Kriegstein A.R. (2016). Expression analysis highlights AXL as a Candidate Zika virus entry receptor in neural stem cells. Cell Stem Cell.

[12] Tang H., Hammack C., Ogden S.C., Wen Z., Qian X., Li Y., Yao B., Shin J., Zhang F., Lee E.M. (2016). Zika virus infects human cortical neural progenitors and attenuates their growth. Cell Stem Cell.

[13] Quicke K.M., Bowen J.R., Johnson E.L., McDonald C.E., Ma H., O’Neal J.T., Rajakumar A., Wrammert J., Rimawi B.H., Pulendran B. (2016). Zika virus infects human placental macrophages. Cell Host Microbe.

[14] Hamel R., Dejarnac O., Wichit S., Ekchariyawat P., Neyret A., Luplertlop N., Perera-Lecoin M., Surasombatpattana P., Talignani L., Thomas F. (2015). Biology of Zika virus infection in human skin cells. J. Virol..

[15] Qian X., Nguyen H.N., Song M.M., Hadiono C., Ogden S.C., Hammack C., Yao B., Hamersky G.R., Jacob F., Zhong C. (2016). Brain-region-specific organoids using mini-bioreactors for modeling ZIKV Exposure. Cell.

[16] Hamel R., Ferraris P., Wichit S., Diop F., Talignani L., Pompon J., Garcia D., Liegeois F., Sall A.A., Yssel H. (2017). African and Asian Zika virus strains differentially induce early antiviral responses in primary human astrocytes. Infect. Genet. Evol..

[17] Meertens L., Labeau A., Dejarnac O., Cipriani S., Sinigaglia L., Bonnet-Madin L., Le Charpentier T., Hafirassou M.L., Zamborlini A., Cao-Lormeau V.M. (2017). Axl Mediates ZIKA virus entry in human glial cells and modulates innate immune responses. Cell Rep..

[18] Van den Pol A.N., Mao G., Yang Y., Ornaghi S., Davis J.N. (2017). Zika virus targeting in the developing brain. J. Neurosci..

[19] Retallack H., Di Lullo E., Arias C., Knopp K.A., Laurie M.T., Sandoval-Espinosa C., Mancia Leon W.R., Krencik R., Ullian E.M., Spatazza J. (2016). Zika virus cell tropism in the developing human brain and inhibition by azithromycin. Proc. Natl. Acad. Sci. USA.

[20] Farina C., Aloisi F., Meinl E. (2007). Astrocytes are active players in cerebral innate immunity. Trends Immunol..

[21] Pedersen I.M., Cheng G., Wieland S., Volinia S., Croce C.M., Chisari F.V., David M. (2007). Interferon modulation of cellular microRNAs as an antiviral mechanism. Nature.

[22] Sarnow P., Jopling C.L., Norman K.L., Schutz S., Wehner K.A. (2006). MicroRNAs: Expression, avoidance and subversion by vertebrate viruses. Nat. Rev. Microbiol..

[23] Tan C.L., Plotkin J.L., Veno M.T., von Schimmelmann M., Feinberg P., Mann S., Handler A., Kjems J., Surmeier D.J., O’Carroll D. (2013). MicroRNA-128 governs neuronal excitability and motor behavior in mice. Science.

[24] Bavia L., Mosimann A.L., Aoki M.N., Duarte Dos Santos C.N. (2016). A glance at subgenomic flavivirus RNAs and microRNAs in flavivirus infections. Virol. J..

[25] Slonchak A., Shannon R.P., Pali G., Khromykh A.A. (2016). Human MicroRNA miR-532-5p exhibits antiviral activity against west Nile virus via suppression of host genes SESTD1 and TAB3 required for virus replication. J. Virol..

[26] Zhu X., He Z., Hu Y., Wen W., Lin C., Yu J., Pan J., Li R., Deng H., Liao S. (2014). MicroRNA-30e* suppresses dengue virus replication by promoting NF-κB-dependent IFN production. PLoS Negl. Trop. Dis..

[27] Ashraf U., Zhu B., Ye J., Wan S., Nie Y., Chen Z., Cui M., Wang C., Duan X., Zhang H. (2016). MicroRNA-19b-3p modulates Japanese encephalitis virus-mediated inflammation via targeting RNF11. J. Virol..

[28] Zhao M., Sun L., Chen S., Li D., Zhang L., He P., Liu X., Zhang L., Zhang H., Yang D. (2015). Borna disease virus infection impacts microRNAs associated with nervous system development, cell differentiation, proliferation and apoptosis in the hippocampi of neonatal rats. Mol. Med. Rep..

[29] Jopling C.L., Yi M., Lancaster A.M., Lemon S.M., Sarnow P. (2005). Modulation of hepatitis C virus RNA abundance by a liver-specific MicroRNA. Science.

[30] Zheng Z., Ke X., Wang M., He S., Li Q., Zheng C., Zhang Z., Liu Y., Wang H. (2013). Human microRNA hsa-miR-296-5p suppresses enterovirus 71 replication by targeting the viral genome. J. Virol..

[31] Medigeshi G.R., Lancaster A.M., Hirsch A.J., Briese T., Lipkin W.I., Defilippis V., Fruh K., Mason P.W., Nikolich-Zugich J., Nelson J.A. (2007). West Nile virus infection activates the unfolded protein response, leading to CHOP induction and apoptosis. J. Virol..

[32] Lanciotti R.S., Lambert A.J., Holodniy M., Saavedra S., Signor Ldel C. (2016). Phylogeny of Zika virus in western Hemisphere, 2015. Emerg. Infect. Dis..

[33] Fonseca K., Meatherall B., Zarra D., Drebot M., MacDonald J., Pabbaraju K., Wong S., Webster P., Lindsay R., Tellier R. (2014). First case of Zika virus infection in a returning Canadian traveler. Am. J. Trop. Med. Hyg..

[34] Sajesh B.V., McManus K.J. (2015). Targeting SOD1 induces synthetic lethal killing in BLM- and CHEK2-deficient colorectal cancer cells. Oncotarget.

[35] Coarfa C., Pichot C., Jackson A., Tandon A., Amin V., Raghuraman S., Paithankar S., Lee A.V., McGuire S.E., Milosavljevic A. (2014). Analysis of interactions between the epigenome and structural mutability of the genome using Genboree Workbench tools. BMC Bioinform..

[36] Riehle K., Coarfa C., Jackson A., Ma J., Tandon A., Paithankar S., Raghuraman S., Mistretta T.A., Saulnier D., Raza S. (2012). The genboree microbiome toolset and the analysis of 16S rRNA microbial sequences. BMC Bioinform..

[37] Vlachos I.S., Zagganas K., Paraskevopoulou M.D., Georgakilas G., Karagkouni D., Vergoulis T., Dalamagas T., Hatzigeorgiou A.G. (2015). DIANA-miRPath v3.0: Deciphering microRNA function with experimental support. Nucleic Acids Res..

[38] Sethupathy P., Corda B., Hatzigeorgiou A.G. (2006). TarBase: A comprehensive database of experimentally supported animal microRNA targets. RNA.

[39] Majer A., Medina S.J., Niu Y., Abrenica B., Manguiat K.J., Frost K.L., Philipson C.S., Sorensen D.L., Booth S.A. (2012). Early mechanisms of pathobiology are revealed by transcriptional temporal dynamics in hippocampal CA1 neurons of prion infected mice. PLoS Pathog..

[40] Major E.O., Miller A.E., Mourrain P., Traub R.G., de Widt E., Sever J. (1985). Establishment of a line of human fetal glial cells that supports JC virus multiplication. Proc. Natl. Acad. Sci. USA.

[41] Smith J.L., Jeng S., McWeeney S.K., Hirsch A.J. (2017). A MicroRNA screen identifies the Wnt signaling pathway as a regulator of the interferon response during flavivirus infection. J. Virol..

[42] Hanners N.W., Eitson J.L., Usui N., Richardson R.B., Wexler E.M., Konopka G., Schoggins J.W. (2016). Western Zika virus in human fetal neural progenitors persists long term with partial cytopathic and limited immunogenic effects. Cell Rep..

[43] Cho H., Shrestha B., Sen G.C., Diamond M.S. (2013). A role for Ifit2 in restricting West Nile virus infection in the brain. J. Virol..

[44] Zheng S., Zhu D., Lian X., Liu W., Cao R., Chen P. (2016). Porcine 2′, 5′-oligoadenylate synthetases inhibit Japanese encephalitis virus replication in vitro. J. Med. Virol..

[45] Tiwari S.K., Dang J., Qin Y., Lichinchi G., Bansal V., Rana T.M. (2017). Zika virus infection reprograms global transcription of host cells to allow sustained infection. Emerg. Microbes Infect..

[46] Dang J., Tiwari S.K., Lichinchi G., Qin Y., Patil V.S., Eroshkin A.M., Rana T.M. (2016). Zika virus depletes neural progenitors in human cerebral organoids through activation of the innate immune receptor TLR3. Cell Stem Cell.

[47] Casseb S.M., Simith D.B., Melo K.F., Mendonca M.H., Santos A.C., Carvalho V.L., Cruz A.C., Vasconcelos P.F. (2016). Drosha, DGCR8, and Dicer mRNAs are down-regulated in human cells infected with dengue virus 4, and play a role in viral pathogenesis. Genet. Mol. Res. GMR.

[48] Bracci-Laudiero L., Celestino D., Starace G., Antonelli A., Lambiase A., Procoli A., Rumi C., Lai M., Picardi A., Ballatore G. (2003). CD34-positive cells in human umbilical cord blood express nerve growth factor and its specific receptor TrkA. J. Neuroimmunol..

[49] Bhattacharyya S., Sen U., Vrati S. (2014). Regulated IRE1-dependent decay pathway is activated during Japanese encephalitis virus-induced unfolded protein response and benefits viral replication. J. Gen. Virol..

[50] Liang Q., Luo Z., Zeng J., Chen W., Foo S.S., Lee S.A., Ge J., Wang S., Goldman S.A., Zlokovic B.V. (2016). Zika virus NS4A and NS4B proteins deregulate Akt-mTOR Signaling in human fetal neural stem cells to inhibit neurogenesis and induce autophagy. Cell Stem Cell.

[51] Barrows N.J., Campos R.K., Powell S.T., Prasanth K.R., Schott-Lerner G., Soto-Acosta R., Galarza-Munoz G., McGrath E.L., Urrabaz-Garza R., Gao J. (2016). A screen of FDA-approved drugs for inhibitors of Zika virus infection. Cell Host Microbe.

[52] McMahon M., Samali A., Chevet E. (2017). Regulation of the unfolded protein response by non-coding RNA. Am. J. Physiol. Cell Physiol..

[53] Scagnolari C., Zingariello P., Vecchiet J., Selvaggi C., Racciatti D., Taliani G., Riva E., Pizzigallo E., Antonelli G. (2010). Differential expression of interferon-induced microRNAs in patients with chronic hepatitis C virus infection treated with pegylated interferon alpha. Virol. J..

[54] Makkoch J., Poomipak W., Saengchoowong S., Khongnomnan K., Praianantathavorn K., Jinato T., Poovorawan Y., Payungporn S. (2016). Human microRNAs profiling in response to influenza A viruses (subtypes pH1N1, H3N2, and H5N1). Exp. Biol. Med..

[55] Thornton G.K., Woods C.G. (2009). Primary microcephaly: Do all roads lead to Rome?. Trends Genet..

[56] Bomont P., Maddox P., Shah J.V., Desai A.B., Cleveland D.W. (2005). Unstable microtubule capture at kinetochores depleted of the centromere-associated protein CENP-F. EMBO J..

[57] Waters A.M., Asfahani R., Carroll P., Bicknell L., Lescai F., Bright A., Chanudet E., Brooks A., Christou-Savina S., Osman G. (2015). The kinetochore protein, CENPF, is mutated in human ciliopathy and microcephaly phenotypes. J. Med. Genet..

[58] Komuro A., Horvath C.M. (2006). RNA- and virus-independent inhibition of antiviral signaling by RNA helicase LGP2. J. Virol..

[59] Faul E.J., Wanjalla C.N., Suthar M.S., Gale M., Wirblich C., Schnell M.J. (2010). Rabies virus infection induces type I interferon production in an IPS-1 dependent manner while dendritic cell activation relies on IFNAR signaling. PLoS Pathog..

[60] So E.Y., Kang M.H., Kim B.S. (2006). Induction of chemokine and cytokine genes in astrocytes following infection with Theiler’s murine encephalomyelitis virus is mediated by the Toll-like receptor 3. Glia.

[61] Nellimarla S., Mossman K.L. (2014). Extracellular dsRNA: Its function and mechanism of cellular uptake. J. Interferon Cytokine Res..

[62] Georgel P., Jiang Z., Kunz S., Janssen E., Mols J., Hoebe K., Bahram S., Oldstone M.B., Beutler B. (2007). Vesicular stomatitis virus glycoprotein G activates a specific antiviral Toll-like receptor 4-dependent pathway. Virology.

[63] Ottino P., Bazan H.E. (2001). Corneal stimulation of MMP-1, -9 and uPA by platelet-activating factor is mediated by cyclooxygenase-2 metabolites. Curr. Eye Res..

[64] Bazan H., Ottino P. (2002). The role of platelet-activating factor in the corneal response to injury. Prog. Retin. Eye Res..

[65] Zhang W.D., Yu X., Fu X., Huang S., Jin S.J., Ning Q., Luo X.P. (2014). MicroRNAs function primarily in the pathogenesis of human anencephaly via the mitogen-activated protein kinase signalling pathway. Genet. Mol. Res..

