# Supplementary material for: MicroRNA and mRNA Dysregulation in Astrocytes Infected with Zika Virus
Source: Viruses. 2017 Oct 14;9(10):297. doi: 10.3390/v9100297 (PMC5691648; doi:10.3390/v9100297)
Supplement: Supplementary file 1 [file viruses-09-00297-s001.pdf]

Supplementary Tables

Supplementary Table 1: Fold-change for miRNAs during Zika virus infection.

miRNA	24 hpi	48 hpi	72 hpi
hsa-miR-17-5p	6.0	1.0	0.2
hsa-miR-146b-5p	3.8	0.6	0.1
hsa-miR-30e-3p	3.3	1.4	0.0
hsa-miR-296-5p	2.6	0.4	0.9
hsa-miR-1303	2.6	0.3	0.7
hsa-miR-4521	2.5	0.7	0.0
hsa-miR-30e-5p	2.4	0.7	0.1
hsa-miR-107	2.2	1.0	0.0
hsa-miR-431-5p	2.1	1.4	0.0
hsa-miR-7-5p	2.1	1.1	0.3
hsa-miR-361-3p	2.0	1.3	0.1
hsa-miR-411-3p	1.2	2.7	0.0
hsa-miR-323a-5p	0.6	2.6	1.4
hsa-miR-194-5p	0.5	2.0	0.0
hsa-miR-9-5p	1.3	1.9	7.2

Supplementary Table 2: miRNA targets at each time point post infection.

Hours Post-Infection	miRNA	No. Potential Genes Targets*	No. Genes after filtering**
24	hsa-miR-17-5p	2186	38
	hsa-miR-146b-5p	501	9
	hsa-miR-30e-3p	1052	21
	hsa-miR-296-5p	450	10
	hsa-miR-1303	221	7
	hsa-miR-4521	150	2
	hsa-miR-30e-5p	1546	32
	hsa-miR-107	2187	32
	hsa-miR-431-5p	0	0
	hsa-miR-7-5p	1830	38
	hsa-miR-361-3p	508	10
48	hsa-miR-411-3p	158	22
	hsa-miR-323a-5p	103	5
	hsa-miR-194-5p	410	45
72	hsa-miR-9-5p	1035	88

* TarBase v7.0 (p-value threshold = 0.05); ** Filtered using downregulated genes at each time point (< or = - 2-fold)

Supplementary Figures

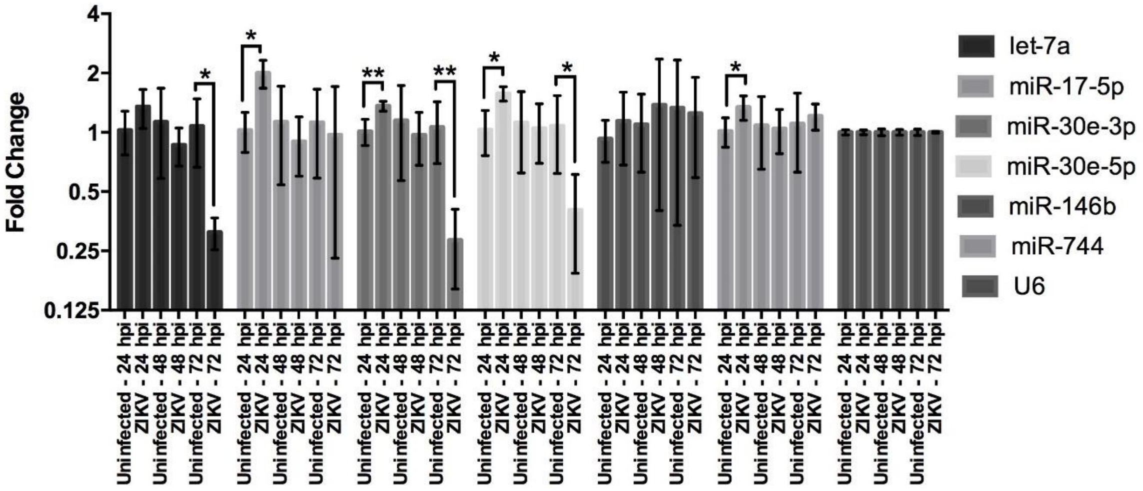

Supplementary Figure 1: Quantitative RT-PCR validation (qPCR), was compared to next generation sequencing expression at 24, 48 and 72 hpi. Validation was performed on three biological replicates and error-bars represent standard deviation. Student's t-test was used to assess significance where * indicates a p-value < 0.05, and ** represents a p-value < 0.005 respectively.

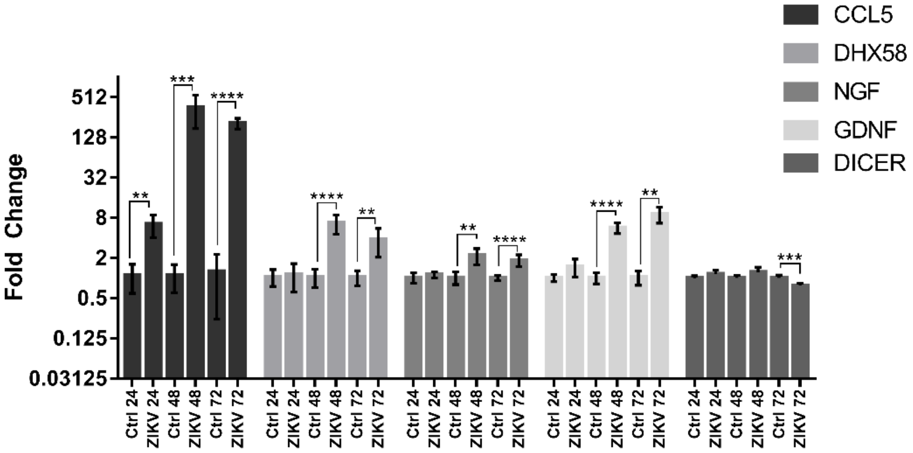

Supplementary Figure 2: Gene expression data in SVG cells. Cells were infected at an MOI of 1 and mRNA was collected 24, 48 and 72 hpi. Transcription of the genes was analyzed by quantitative real-time PCR with GAPDH as a housekeeping gene and expressed as a fold-change compared to uninfected cells (2-ΔΔCt method). Data represent the mean from three replicate experiments, with error bars showing the standard deviation. Samples were compared using Students t-test with significant differences indicated by ** ($p < 0.01$), ***($p < 0.001$), **** ($p < 0.0001$).
